# Effects from a single application of photobiomodulation on pain intensity from perineal trauma related to childbirth: A randomized controlled trial

**DOI:** 10.1002/ijgo.70674

**Published:** 2025-11-19

**Authors:** Edna Jéssica Lima Gondim, Simony Lira Nascimento, Maria Victória Candida Gaitero, Ticiana Aparecida Alves Mira, Andrea Vasconcelos Gonçalves, Fernanda Garanhani Surita

**Affiliations:** ^1^ Department of Obstetrics and Gynecology Universidade Estadual de Campinas Campinas Brazil; ^2^ Physiotherapy Department Federal University of Ceara Fortaleza Brazil

**Keywords:** episiotomy, low‐level light therapy, pain, perineum, postpartum period

## Abstract

**Objective:**

To analyze the effects of a single photobiomodulation (PBM) application on perineal pain and healing in women in the immediate postpartum period.

**Methods:**

Randomized controlled trial, double‐blind, two‐center, with 60 postpartum women with perineal trauma and pain scoring 4 or more on the Numeric Rating Scale (NRS). The experimental group received red light to the lesion and infrared light around it, while the sham group received a simulation. We assessed pain with the NRS and Short‐Form McGill Pain Questionnaire (SF‐MPQ); tissue healing with the REEDA scale (Redness, Edema, Ecchymosis, Discharge, Approximation) at baseline, 30 min (primary outcome), and 12–36 h after intervention and satisfaction with a Likert scale 7–10 days post‐intervention. We conducted bivariate analyses and an analysis of variance for repeated measures.

**Results:**

The experimental and sham groups showed pain reduction with no difference between them for pain scores or healing 30 min post‐intervention (mean ± SD: NRS 3.63 ± 2.57 vs 2.53 ± 2.15; *P* = 0.089; SF‐MPQ 7.83 ± 8.32 vs 5.10 ± 6.42; *P* = 0.108; and REEDA 5.57 ± 3.05 vs 4.47 ± 2.42; *P* = 0.175). Analysis of variance revealed no significant interaction between time and group at any time point evaluated. Of the participants, 84.09% were satisfied or very satisfied with the intervention.

**Conclusions:**

Although both groups experienced a reduction in pain after the intervention, a single PBM application with these parameters was not superior to a sham treatment. Future research could explore multiple applications or different parameters.

**Trial Registration:**

Laser for Pain Relief in Nipple and Perineal Trauma in Postpartum; UTN code U1111‐1279‐3594 (https://ensaiosclinicos.gov.br/rg/RBR‐2qm8jrp/1)

## 1 INTRODUCTION

Perineal trauma affects up to 80% of women who have a vaginal birth, occurring either spontaneously or as a result of an episiotomy.^1^, ^2^ In the early postpartum period, it can lead to pain and delayed healing, which may interfere with daily activities, self‐care, and the ability to care for a newborn. These challenges can significantly affect a woman's quality of life and mental well‐being.^3^, ^4^

Pharmacologic interventions are frequently employed to manage complications resulting from perineal trauma, but non‐pharmacologic approaches offer appealing alternatives. Currently, cryotherapy is the only non‐pharmacologic intervention recommended by the World Health Organization.^5^, ^6^ However, other options—such as photobiomodulation (PBM) therapy—are increasingly being explored.

PBM, or low‐level light therapy, has emerged as a promising treatment. It uses electromagnetic radiation in the red (600–700 nm) and near‐infrared (780–1100 nm) spectra to modulate biologic processes, thereby promoting pain relief, wound healing, and tissue or neural regeneration.^7^, ^8^ PBM stimulates cytochrome c oxidase in mitochondria, enhancing ATP production and reducing inflammation via nitric oxide release and reactive oxygen species. These effects facilitate tissue regeneration and pain relief. Animal studies suggest that PBM may also inhibit nerve conduction in C‐ and A‐delta fibers and modulate neurotransmitter levels, including serotonin and endorphins.^7^, ^9^, ^10^ Hence, we hypothesized that PBM therapy could alleviate perineal pain in postpartum women.

There is a lack of evidence on the use and optimal parameters of PBM for perineal trauma. To address this gap, this study aimed to evaluate the immediate effects of a single application of PBM on pain associated with perineal trauma in the immediate postpartum period. The secondary objectives were to assess perineal healing and self‐perception of tissue healing and to measure women's satisfaction with the intervention.

## 2 MATERIALS AND METHODS

We conducted a randomized, double‐blind, two‐center clinical trial with a 1:1 allocation ratio, within a superiority framework. The Research Ethics Committees of the Universidade Estadual de Campinas and the School Maternity Assis Chateaubriand (Approval numbers 59400922.1.1001.5404 and 9400922.1.3001.5050) approved the study. We collected data from February 1, 2023, to May 20, 2024.

We registered this trial on the Brazilian Registry of Clinical Trials website on January 13, 2023, under UTN code U1111‐1279‐3594, with the public title “Laser for Pain Relief in Nipple and Perineal Trauma in Postpartum” (https://ensaiosclinicos.gov.br/rg/RBR‐2qm8jrp/1). This study is part of a larger research project that evaluated the effects of PBM on perineal and nipple trauma, with the study protocol previously published. In this manuscript, we present the outcomes related to perineal trauma.^11^ We followed the CONSORT Statement.^12^

### 2.1 Setting

We conducted this study at two centers: the Woman's Hospital of Universidade Estadual de Campinas, in southeastern Brazil, and the School Maternity Assis Chateaubriand, affiliated with the Federal University of Ceara, in northeastern Brazil. Both institutions are teaching hospitals and provide services under the Brazilian Unified Health System (SUS).

### 2.2 Participants

Participants were eligible if aged 18 years or older, had a vaginal childbirth with at least an episiotomy or second‐degree perineal laceration, and had a perineal pain score of four or higher on the Numerical Rating Scale for Pain (NRS Pain). Exclusion criteria included active gynecologic infections, puerperal hemorrhage, hearing or communication impairments, bladder catheter use, and tubal ligation within 6 h. The eligibility window was 6–36 h postpartum. All participants signed a written informed consent form before their enrollment in the study. There was no patient or public involvement.

### 2.3 Intervention

**FIGURE 1:**
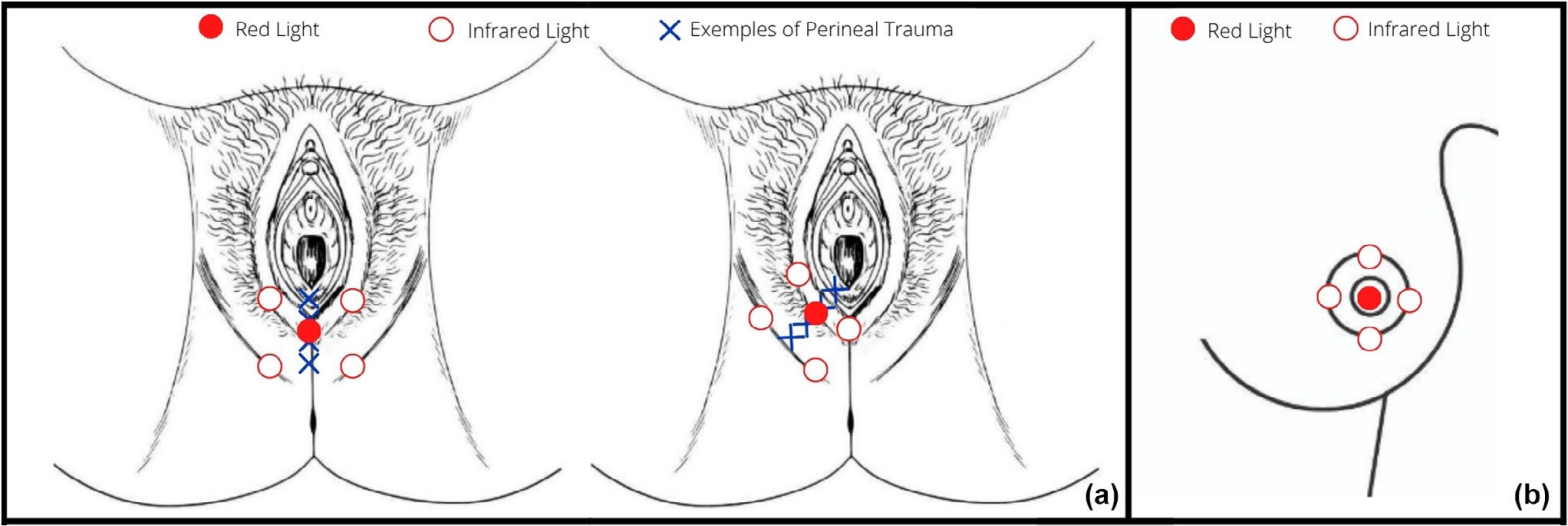
(a) Perineal trauma arm application points demonstration; (b) nipple trauma arm application points demonstration.^11^

Participants in the experimental group received red light therapy (2 J) at one or more points on the perineal trauma site and infrared light therapy (4 J) at four or more points around the site, depending on the trauma extent (Figure 1). The DMC Therapy EC diode laser (100 mW ± 20%), with InGaAlP at 660 ± 10 nm (red) and AlGaAs at 808 ± 10 nm (infrared), was used. Participants could request to stop if discomfort occurred. In the sham group, we performed a simulation without triggering energy. Trained physiotherapists administered the interventions for both groups. Both groups also received routine maternity care. Details regarding the parameters used are shown in Table 1.

**TABLE 1:** Photobiomodulation therapy parameters.

Parameter	Red light	Infrared light
Laser active medium	InGaAlP	AlGaAs
Wavelength,^a^ nm	660 ± 10	808 ± 10
Anatomical site of application	Perineal region	
Power	100 mW ± 20%	
Irradiation time per point, s	20	40
Irradiation area per point, cm^2^	0.04	
Energy per point, J	2	4
Energy density, J/cm^2^	50	100
Number of points treated	≥1 points	≥4 points
Energy per session, K = J	≥2	≥16
Total energy, J	≥2	≥16
Session timing (after childbirth), h	6–36	

^a^ Data are given as mean ± standard deviation.

### 2.4 Outcome measures

To assess perineal pain—our primary outcome—we used the NRS Pain and the Short‐Form McGill Pain Questionnaire (SF‐MPQ).^13^ Secondary outcomes included tissue healing and participant satisfaction. We evaluated healing using the REEDA Scale (Redness, Edema, Ecchymosis, Discharge, and Approximation).^14^ A blinded assessor unaware of group allocation measured outcomes. Reassessment occurred 30 min and 12 h post‐intervention. We assessed self‐perceived healing and satisfaction using a Likert scale.^15^ We systematically monitored adverse effects during the PBM application. Participants recorded pain intensity using the NRS Pain at 1, 3, 6, and 12 h post‐intervention in a pain diary. We conducted a follow‐up telephone interview 7–10 days later to reassess pain (NRS Pain), evaluate healing, and measure satisfaction (Likert scale). Instrument details are available in the study protocol.^11^

### 2.5 Sample size

Sample size was calculated using a mean comparison between two groups, with a significance level of 5% (*α* = 0.05), power of 80% (*β* = 0.20), and mean ± standard deviation (SD) values from the literature.^16^ The experimental group showed a mean pain reduction of 0.4 ± 0.5, whereas the control group showed no change. This corresponds to an effect size of approximately 0.8. A minimum of 52 participants was required to detect a significant difference in perineal pain measured by the NRS. To allow for dropouts, the sample size was increased to 60 women (30 per group).

### 2.6 Randomization

We randomized participants to the experimental group or sham group in a 1:1 ratio using computer‐generated sequences from random.org. To ensure balanced group distribution, we performed block randomization with blocks of 10 participants per center. An unblinded application assistant, not involved in other study procedures, opened sealed, opaque envelopes to maintain allocation concealment.

### 2.7 Statistical analysis

The data collected were stored using the REDCap platform^17^ and subsequently deposited in the Unicamp Data Repository (REDU) to ensure open access. Categorical variables between the sham and experimental groups were compared using the *χ* ^2^ or Fisher exact tests (for expected counts less than 5). Normality of numerical variables was assessed using the Shapiro–Wilk and Kolmogorov–Smirnov tests. Due to non‐normal distribution, numerical variables were compared between groups using the Mann–Whitney *U*‐test for pain and healing outcomes. For comparisons across three or more time points, the Friedman test was used, and for two related time points (before and after), the Wilcoxon signed‐rank test was applied, both due to non‐normality. Those analyses were conducted on an intention‐to‐treat basis and included all 60 participants who were randomly assigned to experimental or sham groups. Additionally, to evaluate the PBM effect over time with women who have all measures (per protocol analysis), we employed Repeated‐measures analysis of variance (ANOVA) on ranked data, followed by Tukey post hoc and contrast profile tests to assess group, time, and interaction effects. No data imputation was performed. SAS version 9.4 was used for statistical analysis. Statistical significance was set at *P* values less than 0.05.

## 3 RESULTS

**FIGURE 2:**
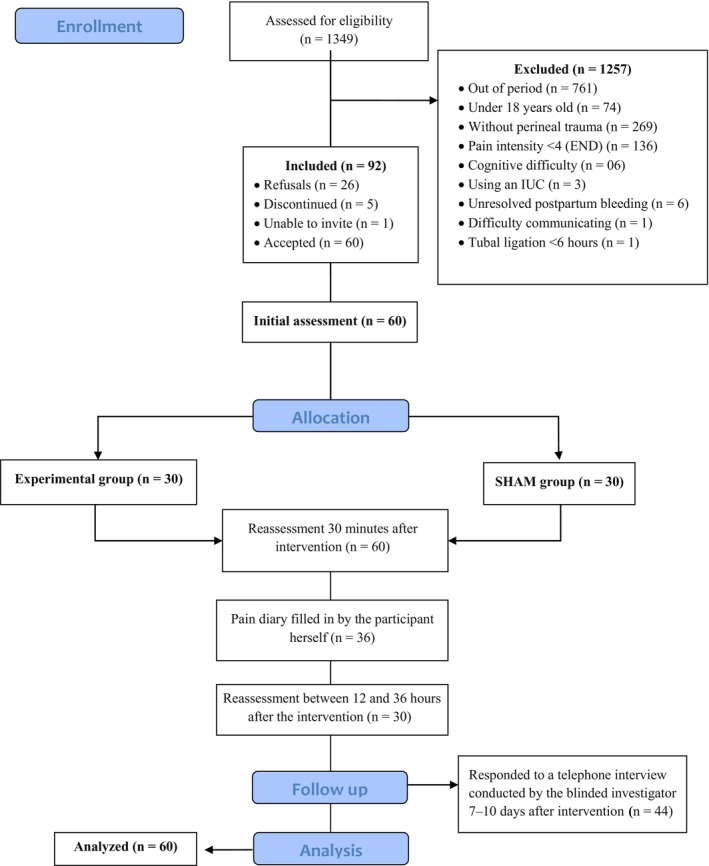
Flowchart CONSORT of participant recruitment and follow up.

We evaluated 60 participants before and 30 min after the intervention. Of these, 36 completed the pain diary (partially or fully), 30 were reassessed between 12 and 36 h post‐intervention, and 44 answered the telephone interview (Figure 2).

The mean age of participants was 25.6 ± 5.1 years. Most self‐identified as having mixed race/ethnicity, had completed or partially completed high school, belonged to a lower socioeconomic stratum, were primiparous, had a mean gestational age at birth of 38.32 ± 1.84 weeks, and sustained second‐degree perineal lacerations. More than half of the sample used postpartum pharmacologic pain relief, administered for a mean of 9 ± 8 h before the start of the study. The groups were homogeneous across sociodemographic, perinatal, and clinical characteristics (Table 2).

**TABLE 2:** Socioeconomic/demographic and clinical/reproductive characteristics of participants.^a^

Characteristics	Total (*n* = 60)	Experimental (*n* = 30)	Sham (*n* = 30)	*P* value
*Socioeconomic/demographic characteristics*				
Age, y	25.57 ± 5.08	26.20 ± 5.14	24.93 ± 5.02	0.432^b^
Self‐reported race/color				
Brown‐skinned	41 (68.33)	20 (66.67)	21 (70.00)	0.324^c^
Black	6 (10.00)	5 (16.67)	1 (3.33)	
White	11 (18.33)	5 (16.67)	6 (20.00)	
Indigenous	1 (1.67)	0 (0.00)	1 (3.33)	
Asian	1 (1.67)	0 (0.00)	1 (3.33)	
Marital status				
Common‐law partnership	29 (48.33)	16 (53.33)	13 (43.33)	0.634^d^
Married	16 (26.67)	8 (26.67)	8 (26.67)	
Single	15 (25.00)	6 (20.00)	9 (30.00)	
Education				
Elementary school level	7 (11.67)	3 (10.0)	4 (13.34)	0.254^c^
High school level	42 (70.00)	22 (73.34)	20 (66.67)	
University level	11 (18.33)	5 (16.67)	6 (20.00)	
Family income (number of minimum wages^e^)				
Up to 2 minimum wages	40 (66.67)	20 (66.67)	20 (66.67)	1.000^c^
Between 2 and 4 minimum wages	17 (28.33)	9 (30.00)	8 (26.67)	
Above 10 minimum wages	3 (5.00)	1 (3.33)	2 (6.67)	
*Obstetric and clinical data*				
Pregestational BMI	24.98 ± 4.93	24.96 ± 5.26	24.99 ± 4.66	0.686^b^
Number of pregnancies	1.57 ± 0.89	1.50 ± 0.90	1.63 ± 0.89	0.443^b^
Number of childbirths	1.40 ± 0.69	1.40 ± 0.72	1.40 ± 0.67	0.866^b^
Number of miscarriages	0.15 ± 0.48	0.10 ± 0.31	0.20 ± 0.61	0.661^b^
Parity				
Primiparous	43 (71.67)	22 (73.33)	21 (70.00)	0.843^c^
Multiparous	17 (28.33)	8 (26.66)	9 (30.00)	
Perineal trauma in previous childbirths				
Episiotomy	6 (10.00)	3 (10.00)	3 (10.00)	1.000^c^
Lacerations	8 (13.33)	3 (10.00)	5 (16.67)	0.707^c^
Didn't happen/remember	2 (3.33)	1 (3.33)	1 (3.33)	1.000^c^
*About last pregnancy/childbirth*				
Gestational weight gain	11.71 ± 6.45	11.39 ± 6.65	12.04 ± 6.33	0.779^b^
Performed perineal massage during pregnancy	2 (3.33)	1 (3.33)	1 (3.33)	1.000^c^
Performed pelvic floor exercises during pregnancy	7 (11.67)	4 (13.33)	3 (10.00)	1.000^c^
Singleton pregnancy	59 (98.33)	29 (96.67)	30 (100.00)	1.000^c^
Spontaneous labor	56 (93.33)	28 (93.33)	28 (93.33)	1.000^c^
Gestational age at childbirth, wk	38.32 (1.84)	38.47 (1.94)	38.17 (1.74)	0.317^b^
Companion during labor	56 (93.33)	28 (93.33)	28 (93.33)	1.000^c^
Pharmacologic analgesia during labor	24 (40.00)	13 (43.33)	11 (36.67)	0.598^d^
Any non‐pharmacologic intervention during labor	28 (46.67)	13 (43.33)	15 (50.00)	0.598^d^
Required forceps	4 (6.67)	4 (13.33)	0 (0.00)	0.112^c^
Required vacuum extractor	4 (6.67)	1 (3.33)	3 (10.00)	0.612^c^
Perineal trauma				
Episiotomy	4 (6.67)	3 (10.00)	1 (3.33)	0.612^c^
Second‐degree laceration	53 (94.64)	25 (92.59)	28 (96.55)	0.605^c^
Third‐degree laceration	3 (5.36)	2 (7.41)	1 (3.45)	
Cephalic fetal presentation	59 (98.33)	29 (96.67)	30 (100.00)	1.000^c^
Newborn weight, g	3137.3 ± 493.96	3045.8 ± 455.13	3228.8 ± 521.47	0.114^b^
Newborn height, cm	48.59 ± 2.31	48.18 ± 2.09	49.02 ± 2.49	0.257^b^
Newborn head circumference, cm	33.85 ± 1.54	33.65 ± 1.75	34.06 ± 1.28	0.486^b^
Use of postpartum analgesics	33 (55.00)	17 (56.57)	16 (53.33)	0.795^d^
Use of postpartum anti‐inflammatory	06 (10.00)	03 (10.00)	03 (10.00)	1.000^c^

Abbreviations: BMI, body mass index (calculated as weight in kilograms divided by the square of height in meters).

^a^ Data are presented as mean ± standard deviation or as number (percentage).

^b^ Mann–Whitney *U*‐test.

^c^ Fisher exact test.

^d^ *χ* ^2^ test.

^e^ The Brazilian minimum wage during the data collection period was approximately equivalent to US$ 263.50.

As shown in Table 3, pain intensity assessed by NRS Pain showed no significant differences between the experimental and sham groups at the pre‐intervention assessment, 30 min post‐intervention, 12–36 h post‐intervention, and 7–10 days post‐intervention. Similarly, we observed no significant differences between the groups in the mean scores of the SF‐MPQ, Present Pain Intensity (PPI) scale, visual analog scale (VAS), and NRS Pain during mobility activities, or in the REEDA score at the initial and three subsequent time points.

**TABLE 3:** Comparison of pain and tissue healing measurements between the experimental and sham groups at different time point assessment (pre‐intervention, 30 min post‐intervention, 12–36 h post‐intervention, 7–10 days post‐intervention).^a^

Instruments	Assessments	Total	Experimental	Sham	*P* value^b^
NRS Pain	Pre‐intervention (*n* = 60)	5.77 ± 1.52	5.50 ± 1.38	6.03 ± 1.63	0.209
	30 min post‐intervention (*n* = 60)	3.08 ± 2.40	3.63 ± 2.57	2.53 ± 2.15	0.089
	12–36 h post‐intervention (*n* = 30)	4.17 ± 2.34	4.07 ± 2.34	4.25 ± 2.41	0.834
	7–10 d post‐intervention (*n* = 44)	1.59 ± 2.15	1.65 ± 2.37	1.52 ± 1.94	0.753
SF‐MPQ score	Pre‐intervention (*n* = 59)	12.22 ± 7.39	13.03 ± 8.21	11.38 ± 6.48	0.438
	30 min post‐intervention (*n* = 59)	6.49 ± 7.51	7.83 ± 8.32	5.10 ± 6.42	0.108
	12–36 hpost‐intervention (*n* = 30)	7.73 ± 5.39	8.93 ± 5.72	6.69 ± 5.03	0.269
VAS	Pre‐intervention (*n* = 60)	4.45 ± 1.74	4.60 ± 2.02	4.30 ± 1.42	0.761
	30 min post‐intervention (*n* = 60)	2.23 ± 2.12	2.64 ± 2.45	1.83 ± 1.68	0.290
	12–36 h post‐intervention (*n* = 30)	2.84 ± 1.96	2.87 ± 1.98	2.81 ± 2.00	1.000
*NRS Pain in mobility*					
Sitting	Pre‐intervention (*n* = 60)	5.62 ± 2.71	5.46 ± 2.85	5.77 ± 2.61	0.672
	30‐min post‐intervention (*n* = 60)	4.10 ± 2.80	4.75 ± 2.78	3.50 ± 2.71	0.091
	12–36 h post‐intervention (*n* = 30)	4.37 ± 2.80	4.64 ± 2.31	4.13 ± 3.22	0.540
Standing	Pre‐intervention (*n* = 60)	5.90 ± 2.86	5.89 ± 2.79	5.90 ± 2.98	0.925
	30‐min post‐intervention (*n* = 60)	4.00 ± 2.93	4.54 ± 3.06	3.50 ± 2.75	0.211
	12‐h post‐intervention (*n* = 30)	4.37 ± 2.80	4.36 ± 2.56	3.31 ± 2.96	0.284
Walking	Pre‐intervention (*n* = 60)	5.95 ± 2.76	6.11 ± 2.76	5.80 ± 2.80	0.623
	30 min post‐intervention (*n* = 60)	3.82 ± 3.07	4.37 ± 3.22	3.33 ± 2.88	0.238
	12–36 h post‐intervention (*n* = 30)	4.41 ± 2.91	4.92 ± 3.01	4.00 ± 2.85	0.413
Lying down (in bed)	Pre‐intervention (*n* = 60)	5.65 ± 2.77	5.36 ± 2.96	5.93 ± 2.59	0.474
	30 min post‐intervention (*n* = 60)	4.22 ± 2.71	4.36 ± 2.96	4.10 ± 2.51	0.759
	12–36 h post‐intervention (*n* = 30)	4.20 ± 2.62	4.21 ± 2.69	4.19 ± 2.64	0.950
REEDA score	Pre‐intervention (*n* = 60)	5.03 ± 2.75	5.53 ± 3.04	4.53 ± 2.37	0.222
	30 min post‐intervention (*n* = 60)	5.02 ± 2.78	5.57 ± 3.05	4.47 ± 2.42	0.175
	12–36 h post‐intervention (*n* = 30)	3.43 ± 3.09	3.57 ± 3.03	3.31 ± 3.24	0.769

Abbreviations: NRS Pain, numeric rating scale for pain; REEDA, Redness, Edema, Ecchymosis, Discharge, and Approximation; SF‐MPQ, Short Form—McGill Pain Questionnaire; VAS, visual analog scale.

^a^ Data are represented as mean ± standard deviation.

^b^ The *P* value related to the Mann–Whitney *U*‐test.

Table 4 presents intragroup comparisons between the pre‐intervention and 30‐min post‐intervention assessments, showing reduced mean pain scores on the NRS, SF‐MPQ, and VAS in both groups; however, no reduction in mean REEDA scores was observed.

**TABLE 4:** Intragroup outcomes of pain and tissue healing for each group at pre‐intervention and 30 min post‐intervention.^a^

Instruments	Experimental (*n* = 30)	Sham (*n* = 30)
NRS Pain		
Pre‐intervention assessment	05.50 ± 1.38	06.03 ± 1.63
30 min post‐intervention	03.63 ± 2.57	02.53 ± 2.13
*P* value (pre vs. 30 min)^b^	<0.001	<0.001
SF‐MPQ score		
Pre‐intervention assessment	13.03 ± 8.21	11.38 ± 6.48
30 min post‐intervention	07.83 ± 8.32	05.10 ± 6.42
*P* value (pre vs. 30 min)^b^	0.001	<0.001
VAS		
Pre‐intervention assessment	04.60 ± 2.02	04.30 ± 1.42
30 min post‐intervention	02.64 ± 2.45	01.83 ± 1.68
*P* value (pre vs. 30 min)^b^	<0.001	<0.001
REEDA score		
Pre‐intervention assessment	05.53 ± 3.04	04.53 ± 2.37
30 min post‐intervention	05.57 ± 3.05	04.47 ± 2.42
*P* value (pre vs. 30 min)^b^	0.655	0.157

Abbreviations: NRS Pain, numeric rating scale for pain; REEDA, Redness, Edema, Ecchymosis, Discharge, and Approximation; SF‐MPQ, Short Form—McGill Pain Questionnaire; VAS, visual analog scale.

^a^ Data are represented as mean ± standard deviation.

^b^ The *P* value refers to the Friedman test for paired samples to compare variables across three or more assessments, followed by the Wilcoxon test for paired samples to compare two time points.

**FIGURE 3:**
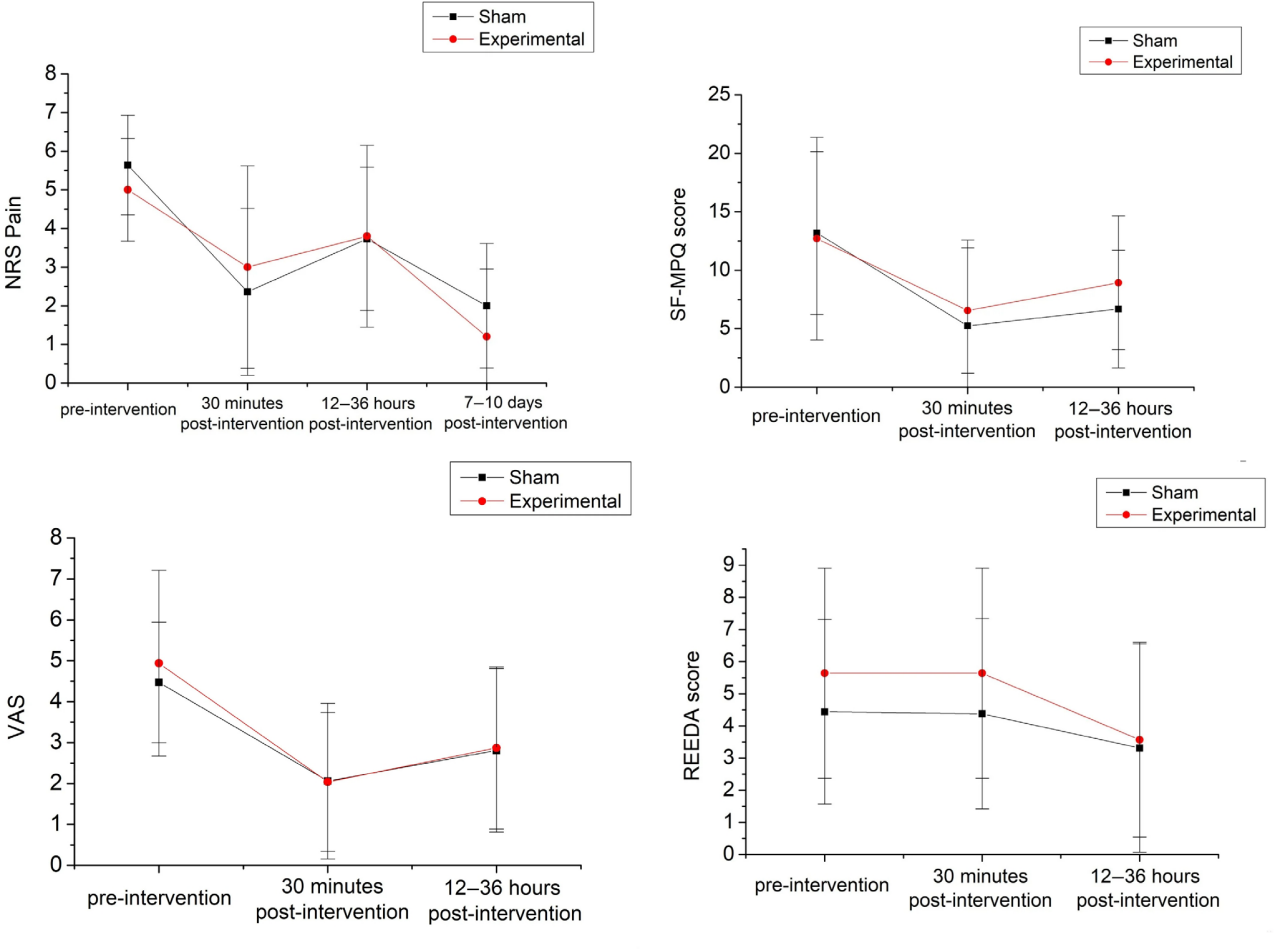
Comparison of outcomes between groups at different assessment time points using four evaluation instruments. NRS, numeric rating scale; REEDA, scale of tissue healing (Redness, Edema, Ecchymosis, Discharge, Approximation); SF‐MPQ, Short‐Form McGill Pain Questionnaire; VAS, visual analog scale.

ANOVA revealed no significant interaction between time and group for NRS Pain scores at any assessment point—pre‐intervention, 30 min, 12–36 h, and 7–10 days post‐intervention (*n* = 21, *P* = 0.328). Similarly, we found no significant differences between the groups at pre‐intervention, 30 min, and 12–36 h post‐intervention for SF‐MPQ (*P* = 0.255), VAS (*P* = 0.943), PPI (*P* = 0.767), or REEDA (*P* = 0.302) scores (*n* = 30) (Figure 3).

We present the frequencies of sensory and affective descriptors from the SF‐MPQ at the pre‐intervention, 30 min post‐intervention, and 12–36 h post‐intervention assessments in the Supporting Information (Table S1). Additionally, we found no significant differences between groups in the frequencies of PPI categories at the same time points (*P* = 0.286; *P* = 0.376; *P* = 0.582, respectively); in the mean NRS Pain scores during self‐care and newborn care activities, or in the mean NRS Pain scores recorded in the pain diary (Table S2).

At the follow‐up interview, participants most frequently selected “marked improvement” as their self‐perceived healing classification in both groups—experimental: 10 (45.45%) versus sham: 16 (76.19%), (*P* = 0.173). Regarding satisfaction, the sham group most often selected “very satisfied”, whereas the experimental group most commonly chose “satisfied” (*P* = 0.004). For adverse effects, only eight participants (13.33%) in the experimental group requested interruption of the intervention because of sensations of heat, pain, or discomfort (*P* = 0.005) (Table S2).

## 4 DISCUSSION

We observed no significant differences between the experimental and sham groups in pain relief or tissue healing improvement, within the parameters of this trial, following a single application of PBM for treating perineal birth‐related trauma. Furthermore, both groups demonstrated a significant reduction in pain intensity over time.

We can hypothesize reasons that may have contributed to the limited effect on pain relief. One possibility is that the 4 J dose of infrared light may not have been optimal. We selected this dosage because it is significantly higher than doses tested in previous trials, yet still falls within the therapeutic range suggested in the literature.^18^

Previous randomized controlled trials investigating PBM for pain relief related to perineal trauma also found no significant differences between the experimental and sham groups. One study applied a single treatment using a device with a power of 35 mW and a total energy of 1.05 J, administered at three points with simultaneous red and infrared light.^19^ Another study conducted three applications using a device with the same power (35 mW) and a total energy of 5.4 J, applied at nine points, also using red and infrared light simultaneously.^16^ The most recent study also evaluated tissue healing and found no differences between the groups in the REEDA score or its components across the four assessments.^16^ In these studies, the devices used had lower power outputs, and the administered doses were similarly minimal.

A recent clinical trial in Brazil (*n* = 56) compared PBM (3 J/cm^2^ red light for 30 seconds per point and 6 J/cm^2^ infrared light for 60 seconds per point) with cryotherapy (crushed ice in a latex glove for 20 min). The PBM group showed significantly more pronounced pain reduction and better tissue healing than the cryotherapy group, both immediately and 24 h after treatment, based on VAS, SF‐MPQ, and REEDA scores (*P* < 0.05). However, the study was unblinded and lacked a control or sham group, which limits the strength of its conclusions.^20^ Hence, PBM effectiveness appears dose‐dependent, highlighting the need for well‐designed clinical trials to establish optimal parameters.^21^

Additionally, a single application may not have been sufficient to achieve clinically significant pain reduction. However, this approach was feasible given the recommended 24‐h minimum interval between PBM applications, as its effects may last up to 24 h, and reducing the interval could result in dose overlap.^22^ In most Brazilian hospitals, postpartum women without clinical complications are typically discharged within 24–48 h following vaginal childbirth, making repeated applications in a hospital environment unfeasible. Considering that perineal trauma is often not superficial, it is plausible that a series of PBM applications and more rigorous monitoring are required to observe meaningful improvements in pain and healing.

Another relevant finding of our study was that more than half of the participants had used medication for pain relief postpartum. This finding raises particular concern for the target population because of the potential adverse effects of pharmacologic pain relief on the mother and the breastfeeding newborn.^6^, ^23^, ^24^ These observations highlight the importance of identifying a non‐pharmacologic intervention that could effectively address pain relief without these risks. It is worth noting that, despite using these medications, the participants' complaints appeared unresolved, as they had to report at least moderate pain score (NRS Pain ≥ 4) to be included in the study.

The main limitation of our study was the extended period over which participants could receive PBM, spanning more than one day. However, this approach was necessary due to the context of public hospitals, where postpartum women do not always move immediately to rooming‐in care while waiting for available beds. Furthermore, we experienced a loss of participants who completed the pain diary and responded to the telephone interview, which we might attribute to the demanding routine during the first days postpartum. Courtesy bias and the Hawthorne effect may have influenced responses in the sham group, as participants might have reported positive outcomes due to perceived expectations or the attention received during the study. Inclusion of a third group receiving no intervention could help to mitigate these biases and allow a more accurate comparison of treatment effects.

On the other hand, we highlight the originality of our study, as it is the first to use higher‐power devices compared with previous studies and to test higher doses commonly applied in clinical practice. Our study was well‐designed and two‐center, including women from two different regions of Brazil. We chose to compare the experimental group with a sham group to assess the actual effect of PBM therapy. Additionally, the participants and the evaluators were blinded, minimizing potential biases. Our findings are valuable for guiding future research. Such studies may help refine the intervention protocol and establish the ideal and safe therapeutic dose for these cases. We believe in the potential of this treatment because it can be delivered quickly, involves minimal to no adverse effects, and enables the treatment of multiple individuals with a single device.

In conclusion, a single application of PBM, using the parameters selected in our study, did not result in significant differences between the experimental and sham groups in terms of pain relief or enhanced healing. The experimental and control groups experienced improvements in these aspects over time, likely due to natural healing processes. Even so, most participants reported moderate to high satisfaction with the intervention. There is a need for more well‐designed studies to explore the effects of multiple applications or alternative PBM parameters on perineal trauma to determine the optimal dosage for this non‐pharmacologic intervention.

## AUTHOR CONTRIBUTIONS

EJLG, SLN, MVCG, TAAM, AVG, and FGS made substantial contributions to the conception and design of the study. EJLG, MVCG, and TAAM were responsible for data collection. SLN, TAAM, and FGS provided statistical expertise in the clinical trial design. EJLG drafted the initial version of the manuscript. All authors critically reviewed the manuscript, approved the final version, and accepted full responsibility for the integrity of the work.

## FUNDING INFORMATION

The authors have not declared a specific grant for this research from any funding agency in the public, commercial, or not‐for‐profit sectors.

## CONFLICT OF INTEREST STATEMENT

The authors have no conflicts of interest.

## Supporting information


**Table S1.** Sensory and affective descriptors of the SF‐MPQ at the three assessment time points.
**Table S2.** Frequencies of present pain intensity descriptors at the three assessment time points; mean NRS Pain scores during self‐care, newborn care, and from the pain diary; and frequencies of self‐perceived tissue healing, satisfaction, and adverse effects.

## Data Availability

Unicamp Research Data Repository (https://doi.org/10.25824/redu/RVQYWJ).
