# Supplementary material for: Effects from a single application of photobiomodulation on pain intensity from perineal trauma related to childbirth: A randomized controlled trial
Source: Int J Gynaecol Obstet. 2025 Nov 19;173(2):808–17. doi: 10.1002/ijgo.70674 (PMC13094685; doi:10.1002/ijgo.70674)
Supplement: Supplementary file 1 — Table S1. Sensory and affective descriptors of the SF‐MPQ at the three assessment time points. Table S2. Frequencies of present pain intensity descriptors at the three assessment time points; mean NRS Pain scores during self‐care, newborn care, and from the pain diary; and frequencies of self‐perceived tissue healing, satisfaction, and adverse effects. [file IJGO-173-808-s001.zip › Supplementary file.docx]

Table S1 – Sensory and Affective Descriptors of the SF-MPQ at the Three Assessment Time Points.

| **SF-MPQ descriptors** | **Assessments** | **Intensity classification**  **n (%)** | | | |
| --- | --- | --- | --- | --- | --- |
| Throbbing | Pre-intervention (n = 59) | **None** | **Mild** | **Moderate** | **Severe** |
|  |  | 39 (66.10) | 07 (11.86) | 11 (18.64) | 02 (03.39) |
|  | 30 - min post-intervention (n = 59) | 47 (79.66) | 04 (06.78) | 08 (13.56) | 00 (00.00) |
|  | 12 - 36 hours post-intervention (n = 30) | 22 (73.33) | 05 (16.67) | 03 (10.00) | 00 (00.00) |
|  | | | | | |
| Shooting | Pre-intervention (n = 59) | 27 (45.76) | 15 (25.42) | 16 (27.12) | 01 (01.69) |
|  | 30 - min post-intervention (n = 59) | 42 (71.19) | 10 (16.95) | 07 (11.86) | 00 (00.00) |
|  | 12 - 36 hours post-intervention (n = 30) | 21 (70.00) | 04 (13.33) | 05 (16.67) | 00 (00.00) |
|  | | | | | |
| Stabbing | Pre-intervention (n = 59) | 50 (84.75) | 02 (03.39) | 04 (06.78) | 03 (05.08) |
|  | 30 - min post-intervention (n = 59) | 49 (83.05) | 05 (08.47) | 04 (06.78) | 01 (01.69) |
|  | 12 - 36 hours post-intervention (n = 30) | 27 (90.00) | 02 (06.67) | 01 (03.33) | 00 (00.00) |
|  | | | | | |
| Sharp | Pre-intervention (n = 59) | 28 (47.46) | 13 (22.03) | 13 (22.03) | 05 (08.47) |
|  | 30 - min post-intervention (n = 59) | 38 (64.41) | 12 (20.34) | 06 (10.17) | 03 (05.08) |
|  | 12 - 36 hours post-intervention (n = 30) | 21 (70.00) | 04 (13.33) | 05 (16.67) | 00 (00.00) |
|  | | | | | |
| Cramping | Pre-intervention (n = 59) | 18 (30.51) | 17 (28.81) | 19 (32.20) | 05 (08.47) |
|  | 30 - min post-intervention (n = 59) | 39 (66.10) | 07 (11.86) | 11 (18.64) | 02 (03.39) |
|  | 12 - 36 hours post-intervention (n = 30) | 15 (50.00) | 08 (26.67) | 06 (20.00) | 01 (03.33) |
|  | | | | | |
| Gnawing | Pre-intervention (n = 59) | 16 (27.12) | 11 (18.64) | 29 (49.15) | 03 (05.08) |
|  | 30 - min post-intervention (n = 59) | 36 (61.02) | 12 (20.34) | 09 (15.25) | 02 (03.39) |
|  | 12 - 36 hours post-intervention (n = 30) | 15 (50.00) | 07 (23.33) | 08 (26.67) | 00 (00.00) |
|  | | | | | |
| Hot/Burning | Pre-intervention (n = 59) | 36 (61.02) | 05 (08.47) | 11 (18.64) | 07 (11.86) |
|  | 30 - min post-intervention (n = 59) | 47 (79.66) | 05 (08.47) | 04 (06.78) | 03 (05.08) |
|  | 12 - 36 hours post-intervention (n = 30) | 18 (60.00) | 06 (20.00) | 05 (16.67) | 01 (03.33) |
|  | | | | | |
| Aching | Pre-intervention (n = 59) | 09 (15.25) | 12 (20.34) | 30 (50.85) | 08 (13.56) |
|  | 30 - min post-intervention (n = 59) | 23 (38.98) | 17 (28.81) | 16 (27.12) | 03 (05.08) |
|  | 12 - 36 hours post-intervention (n = 30) | 07 (23.33) | 09 (30.00) | 12 (40.00) | 02 (06.67) |
|  | | | | | |
| Heavy | Pre-intervention (n = 59) | 34 (57.63) | 08 (13.56) | 15 (25.42) | 02 (03.39) |
|  | 30 - min post-intervention (n = 59) | 45 (76.27) | 10 (16.95) | 03 (05.08) | 01 (01.69) |
|  | 12 - 36 hours post-intervention (n = 30) | 21 (70.00) | 06 (20.00) | 03 (10.00) | 00 (00.00) |
|  | | | | | |
| Tender | Pre-intervention (n = 59) | 10 (16.95) | 12 (20.34) | 22 (37.29) | 15 (25.42) |
|  | 30 - min post-intervention (n = 59) | 27 (45.76) | 13 (22.03) | 17 (28.81) | 02 (03.39) |
|  | 12 - 36 hours post-intervention (n = 30) | 11 (36.67) | 05 (16.67) | 12 (40.00) | 02 (06.67) |
|  | | | | | |
| Spliting | Pre-intervention (n = 59) | 09 (66.10) | 05 (08.47) | 11 (18.64) | 04 (06.78) |
|  | 30 - min post-intervention (n = 59) | 50 (84.75) | 07 (11.86) | 00 (00.00) | 02 (03.39) |
|  | 12 - 36 hours post-intervention (n = 30) | 25 (83.33) | 02 (06.67) | 02 (06.67) | 01 (03.33) |
|  | | | | | |
| Tiring/Exhausting | Pre-intervention (n = 59) | 36 (61.02) | 08 (13.56) | 13 (22.03) | 02 (03.39) |
|  | 30 - min post-intervention (n = 59) | 44 (74.58) | 06 (10.17) | 08 (13.56) | 01 (01.69) |
|  | 12 - 36 hours post-intervention (n = 30) | 20 (66.67) | 05 (16.67) | 05 (16.67) | 00 (00.00) |
|  | | | | | |
| Sickening | Pre-intervention (n = 59) | 56 (94.92) | 01 (01.69) | 01 (01.69) | 00 (00.00) |
|  | 30 - min post-intervention (n = 59) | 58 (98.31) | 01 (01.69) | 00 (00.00) | 00 (00.00) |
|  | 12 - 36 hours post-intervention (n = 30) | 29 (96.67) | 00 (00.00) | 01 (03.33) | 00 (00.00) |
|  | | | | | |
| Fearful | Pre-intervention (n = 59) | 42 (71.19) | 09 (15.25) | 05 (08.47) | 03 (05.08) |
|  | 30 - min post-intervention (n = 59) | 49 (83.05) | 04 (06.78) | 06 (10.17) | 00 (00.00) |
|  | 12 - 36 hours post-intervention (n = 30) | 27 (90.00) | 01 (03.33) | 01 (03.33) | 01 (03.33) |
|  | | | | | |
| Punishing/Cruel | Pre-intervention (n = 59) | 50 (84.75) | 05 (08.47) | 03 (05.08) | 01 (01.69) |
|  | 30 - min post-intervention (n = 59) | 53 (89.83) | 01 (01.69) | 04 (06.78) | 01 (01.69) |
|  | 12 - 36 hours post-intervention (n = 30) | 28 (93.33) | 01 (03.33) | 01 (03.33) | 00 (00.00) |

Table S2 - Frequencies of Present Pain Intensity Descriptors at the Three Assessment Time Points; Mean NRS Pain Scores During Self-Care, Newborn Care, and from the Pain Diary; and Frequencies of Self-Perceived Tissue Healing, Satisfaction, and Adverse Effects.

| **Present Pain Intensity** | | | | | |
| --- | --- | --- | --- | --- | --- |
| **Assessments** | **Classification** | **Total**  **n (%)** | **Experimental**  **n (%)** | **Sham**  **n (%)** | **p-value^a^** |
| Pre-intervention  (n = 60) | No pain | 18 (30.00) | 07 (23.33) | 11 (36.67) | 0.286 |
|  | Mild | 16 (26.67) | 08 (26.67) | 08 (26.67) |  |
|  | Discomforting | 23 (38.33) | 14 (46.67) | 09 (30.00) |  |
|  | Distressing | 02 (03.33) | 00 (00.00) | 02 (06.67) |  |
|  | Horrible | 01 (01.67) | 01 (03.33) | 00 (00.00) |  |
|  | Excruciating | 00 (00.00) | 00 (00.00) | 00 (00.00) |  |
|  |  |  |  |  |  |
| 30 - min  post-intervention  (n = 60) | No pain | 27 (45.00) | 12 (40.00) | 15 (50.00) | 0.376 |
|  | Mild | 22 (36.67) | 10 (33.33) | 12 (40.00) |  |
|  | Discomforting | 10 (16.67) | 07 (23.33) | 03 (10.00) |  |
|  | Distressing | 00 (00.00) | 00 (00.00) | 00 (00.00) |  |
|  | Horrible | 00 (00.00) | 00 (00.00) | 00 (00.00) |  |
|  | Excruciating | 01 (01.67) | 01 (03.33) | 00 (00.00) |  |
|  |  |  |  |  |  |
| 12 - 36 hours  post-intervention  (n = 30) | No pain | 09 (30.00) | 04 (28.57) | 05 (31.25) | 0.582 |
|  | Mild | 13 (43.33) | 05 (35.71) | 08 (50.00) |  |
|  | Discomforting | 08 (26.67) | 05 (35.71) | 03 (18.75) |  |
|  | Distressing | 00 (00.00) | 00 (00.00) | 00 (00.00) |  |
|  | Horrible | 00 (00.00) | 00 (00.00) | 00 (00.00) |  |
|  | Excruciating | 00 (00.00) | 00 (00.00) | 00 (00.00) |  |
| **NRS Pain on activities on pre-intervention assessment** | | | | | |
| **Activities** | | **Total**  **mean (SD)** | **Experimental**  **mean (SD)** | **Sham**  **mean (SD)** | **p-value^b^** |
| Self-care  (n = 60) | Urination | 4.89 (3.23) | 4.65 (3.31) | 5.10 (3.20) | 0.649 |
|  | Evacuation | 5.00 (7.07) | - | - | - |
|  | Bathing | 5.23 (3.04) | 5.00 (3.16) | 5.47 (2.96) | 0.704 |
|  | Eating | 0.60 (1.66) | 0.93 (2.08) | 0.27 (1.01) | 0.115 |
|  | Sleeping | 2.67 (3.24) | 3.61 (3.49) | 1.60 (2.62) | **0.049** |
| Newborn care  (n = 60) | Breastfeeding | 1.82 (2.80) | 1.71 (2.85) | 1.92 (2.80) | 0.760 |
|  | Change diapers | 2.16 (2.93) | 2.13 (2.39) | 2.18 (3.41) | 0.647 |
| **Pain Diary** (n = 36) | | | | | |
| **Time after intervention** | | **Total**  **mean (SD)** | **Experimental**  **mean (SD)** | **Sham**  **mean (SD)** | **p-value^b^** |
| 1 hour | | 4.17 (2.30) | 4.71 (2.37) | 3.68 (2.19) | 0.206 |
| 3 hours | | 3.84 (2.35) | 4.53 (2.58) | 3.25 (2.02) | 0.127 |
| 6 hours | | 4.00 (2.51) | 4.44 (2.53) | 3.61 (2.50) | 0.390 |
| 12 hours | | 4.55 (3.16) | 5.27 (3.06) | 3.88 (3.20) | 0.197 |
| **Self-perception of tissue healing** (n = 43) | | | | | |
| **Classification** | | **Total**  **n (%)** | **Experimental**  **n (%)** | **Sham**  **n (%)** | **p-value^b^** |
| **Worsened** | | 02 (04.65) | 01 (04.55) | 01 (04.76) | 0.173 |
| **No change** | | 02 (04.65) | 02 (09.09) | 00 (00.00) |  |
| **Slight improvement** | | 11 (25.58) | 08 (36.36) | 03 (14.29) |  |
| **Marked improvement** | | 26 (60.47) | 10 (45.45) | 16 (76.19) |  |
| **Completely healed** | | 02 (04.65) | 01 (04.55) | 01 (04.76) |  |
| **Satisfaction** (n = 44) | | | | | |
| **Classification** | | **Total**  **n (%)** | **Experimental**  **n (%)** | **Sham**  **n (%)** | **p-value^b^** |
| Very satisfied | | 21 (47.73) | 06 (26.09) | 15 (71.43) | **0.004** |
| Satisfied | | 16 (36.36) | 10 (43.48) | 06 (28.57) |  |
| Neither satisfied nor dissatisfied | | 05 (11.36) | 05 (21.74) | 00 (00.00) |  |
| Dissatisfied | | 02 (04.55) | 02 (08.70) | 00 (00.00) |  |
| Very dissatisfied | | 00 (00.00) | 00 (00.00) | 00 (00.00) |  |
| **Adverse effects** (n = 60) | | | | | |
|  | | **Total**  **n (%)** | **Experimental**  **n (%)** | **Sham**  **n (%)** | **p-value^b^** |
| Yes | | 08 (13.33) | 08 (26.67) | 00 (00.00) | **0.005** |
| No | | 52 (86.67) | 22 (73.33) | 30 (100.0) |  |

^a^ Fishers Exact Test; ^b^ Mann-Whitney Test.
